# Correlation Between Psoas and Diaphragmatic Ultrasound Indices for the Assessment of Sarcopenia in Patients with Inflammatory Bowel Disease: A Prospective Single-Center Study

**DOI:** 10.3390/nu18040622

**Published:** 2026-02-13

**Authors:** Chiara Maria Palmisano, Paola Dell’Aquila, Antonella Contaldo, Giuseppe Losurdo, Mariabeatrice Principi

**Affiliations:** Department of Precision and Regenerative Medicine and Ionian Area (DiMePRe-J), University of Bari “Aldo Moro”, 70100 Bari, Italy

**Keywords:** inflammatory bowel disease, sarcopenia, malnutrition, ultrasound, quality of life

## Abstract

**Background and Aim**: Sarcopenia is increasingly recognized as a clinically significant complication of inflammatory bowel disease (IBD), influencing both medical management and surgical outcomes. Accurate and accessible diagnostic tools are essential for assessing muscle mass and function in this population. The iliopsoas (IP) muscle has traditionally been used as a marker of sarcopenia, but its deep anatomical location requires skilled operators. Conversely, the diaphragm (DM), being more superficial, may serve as a more feasible surrogate. This study aimed to assess the correlation between IP- and DM-derived ultrasound indices in patients with IBD and to explore their association with sarcopenia risk. Methods: This prospective single-center study enrolled 353 IBD patients (Crohn’s disease [CD] and ulcerative colitis [UC]). Overall, 57 patients had a SARC-F score ≥ 4 and underwent intestinal ultrasound (US). The transverse diameter of the right IP muscle was measured in triplicate, and the psoas-to-height ratio (PMTH, mm/m) was calculated. Diaphragm thickness was assessed during inspiration and expiration, and the diaphragm-to-height ratio was derived. Pearson correlation, Bland–Altman analysis and multivariable regression (adjusted for age and sex) were performed to test associations. Results: The mean IP diameter was 28.40 mm (28.82 mm in males, 27.02 mm in females), with a mean PMTH of 16.62 mm/m. Diaphragm thickness was 20.4 ± 5.0 mm at inspiration and 10.7 ± 3.5 mm at expiration, yielding a mean difference of 9.7 ± 3.4 mm. The diaphragm-to-height ratio was 0.59 ± 0.21 mm/m. Pearson correlation revealed a moderate positive association between PMTH and the diaphragm index (r = 0.3568, *p* < 0.05). Bland–Altman analysis disclosed a symmetrical distribution. Multivariable regression confirmed that the diaphragm index increased linearly with PMTH (β = 0.018, 95% CI 0.005–0.030; *p* = 0.008). Neither age nor sex significantly affected the results. Conclusions: Muscle ultrasound is a reliable and reproducible method for evaluating sarcopenia in IBD. The diaphragm, due to its superficial anatomical location and ease of measurement, shows a significant correlation with psoas muscle parameters and may serve as a practical surrogate marker in clinical practice. Larger multicenter studies are warranted to validate these findings.

## 1. Introduction

Inflammatory bowel disease (IBD) is a chronic, relapsing inflammatory disorder with systemic manifestations that extend beyond the gastrointestinal tract. Among these, skeletal muscle health has gained increasing attention, as sarcopenia is strongly associated with adverse clinical outcomes, including prolonged or recurrent hospitalizations, higher postoperative complication rates, and therapeutic failure [1,2,3]. Sarcopenia, defined as the progressive loss of muscle mass, strength, and function [4], was initially considered a condition confined to the elderly (primary sarcopenia). However, growing evidence indicates that secondary sarcopenia may develop earlier in patients with chronic diseases, including IBD [5]. Prevalence studies have reported that up to 12% of IBD patients with a mean age of 31 years are sarcopenic, with rates rising to 52% in specific cohorts of Crohn’s disease (CD) patients [6]. Multiple mechanisms contribute to sarcopenia in IBD, including persistent systemic inflammation, impaired protein absorption, reduced dietary intake, chronic corticosteroid exposure, and physical inactivity [7]. Although malnutrition is a recognized risk factor, a substantial proportion of sarcopenic IBD patients are paradoxically overweight or obese [8], underscoring the complexity of identifying sarcopenia in this population.

The European Society for Clinical Nutrition and Metabolism (ESPEN) guidelines recommend systematic screening for malnutrition and sarcopenia both at diagnosis and during follow-up [9]. However, gold-standard imaging techniques such as magnetic resonance imaging (MRI) and computed tomography (CT) are limited in routine practice due to high costs, restricted availability, radiation exposure, and the need for specialized operators [4].

Alternative tools, including dual-energy X-ray absorptiometry (DXA) and bioelectrical impedance analysis (BIA), are more accessible and are commonly used in clinical practice. Nevertheless, their applicability to IBD remains uncertain, as validated disease-specific cut-offs are lacking and neither provides information about muscle quality [10].

Intestinal ultrasound (US) has recently emerged as a promising bedside method for evaluating sarcopenia in IBD. It is non-invasive, inexpensive, portable, and capable of providing both quantitative and qualitative assessments of muscle structure and function [11]. The SARCopenia through UltraSound (SARCUS) working group has standardized anatomical landmarks and acquisition protocols, improving reproducibility across studies [11]. Compared with CT and DXA, ultrasound has demonstrated comparable or even superior accuracy in estimating muscle mass [12], and US-derived indices have been shown to predict hospitalization risk and mortality [13].

Over the past decade, research has focused on cross-sectional imaging of muscles such as the psoas and erector spinae, from which several indices have been derived, including the psoas muscle index (PMI), psoas muscle thickness-to-height ratio (PMTH), psoas muscle area (PMA), and skeletal muscle index (SMI) [14,15]. Although these CT-based parameters are validated prognostic tools, CT is not suitable for point-of-care use and is limited by radiation exposure and cost [16].

To overcome these limitations, Tandon et al. proposed using ultrasound-based thigh muscle measurements as surrogates for sarcopenia, although this approach has yet to be validated against clinical outcomes [14]. Japanese studies have demonstrated that ultrasound-based psoas measurements correlate well with CT in both healthy individuals and patients with cirrhosis [12,17]. However, no comparable data exist for European IBD populations, and psoas ultrasound remains technically challenging due to the muscle’s depth and the need for operator expertise.

In contrast, diaphragm ultrasound offers several advantages. It is a rapid, reproducible, and safe technique with a relatively short learning curve and can be performed at the bedside using standard ultrasound equipment. In less than 15 min, it provides reliable morphological and functional information [18]. Despite these advantages, diaphragm ultrasound has not yet been validated as a predictive tool for sarcopenia in IBD. Given these considerations, rapid ultrasound-based screening for sarcopenia in IBD could facilitate timely nutritional and rehabilitative interventions. Previous studies have proposed that a height-corrected psoas thickness < 16.8 mm/m represents a diagnostic threshold for sarcopenia, with higher cut-offs reported in men compared with women [19].

## 2. Materials and Methods

### 2.1. Study Design and Setting

This is prospective, single center, cohort study aimed to evaluate the feasibility and diagnostic accuracy of diaphragmatic muscle ultrasound compared with psoas muscle ultrasound, and to determine their correlation with the clinical sarcopenia index (SARC-F score). The primary objective was to investigate the correlation between the SARC-F score and ultrasound assessment of the psoas muscle in patients with IBD. The secondary objective assesses the direct correlation between psoas and diaphragmatic muscle indices.

The study enrolled adult patients with histologically confirmed IBD who were undergoing intestinal ultrasound (Esaote S.p.A Italy), primarily for routine monitoring. The eligibility required SARC-F score ≥ 4, indicating suspected sarcopenia. All of participants underwent both psoas (IP) and diaphragm (DM) ultrasound examinations and completed the SARC-F questionnaire. Demographic, clinical, and treatment-related data were collected, including past and current medical history. IBD diagnosis has been established according to the European Crohn’s and Colitis Organisation (ECCO) guidelines, integrating clinical, biochemical, endoscopic, and histological findings. Patients have been classified according to the Montreal classification system and for disease activity was used the Harvey–Bradshaw Index (HBI) for CD, and for UC the Partial Mayo Score. Exclusion criteria included contraindications to ultrasound examination, severe cognitive impairment, or inability to provide informed consent. Corticosteroid use was defined as ≥20 mg/day methylprednisolone (or equivalent) for ≥2 weeks within the past 12 months.

At the enrolment nutritional status, sarcopenia risk and muscles ultrasound examination were assessed.

Nutritional status was evaluated using body mass index (BMI), in line with World Health Organization (WHO) recommendations, and with the Malnutrition Universal Screening Tool (MUST), endorsed by ESPEN and the Global Leadership Initiative on Malnutrition (GLIM) [20].

The MUST score assesses three parameters: (i) BMI, (ii) unintentional weight loss in the preceding 3–6 months, and (iii) the presence of any acute illness causing inadequate nutritional intake for ≥5 days. The total score ranges from 0 to 2, corresponding to low (0), medium (1), or high (≥2) risk of malnutrition.

Sarcopenia risk was screened using the SARC-F questionnaire, a validated self-reported tool assessing five components: strength, assistance in walking, rising from a chair, climbing stairs, and falls. Each item is scored 0–2, for a total score range of 0–10. A score ≥ 4 was considered indicative of sarcopenia risk [21].

All muscle ultrasound examinations were performed with a diagnostic device equipped with a convex probe (3.5–5 MHz) and a linear probe (18–25 MHz) (Esaote S.p.A Italy). Two independent operators, blinded to each other’s results and to clinical and laboratory data, conducted all measurements to ensure accuracy and to evaluate inter- and intra-observer variability. Measurements followed the recommendations of the SARCopenia through UltraSound (SARCUS) working group [11].

**Figure 1:**
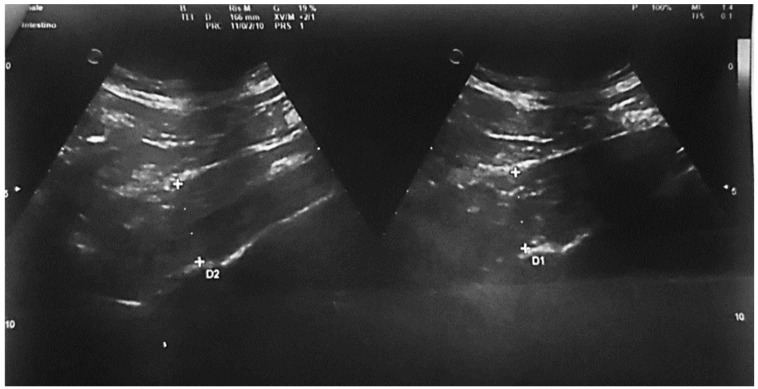
Muscle US measurement. Right psoas muscle and concomitantly lying lumbar vertebrae were accurately recognized in each patient. Abdominal probe was positioned in a sagittal plane subphrenally right and just above the upper anatomical limit of the pelvis (iliac crest). Sliding the ultra-sonic probe in the anteromedial to posterolateral direction, the largest ultrasonically visible psoas muscle diameter was defined.

Psoas muscle (IP): the right psoas and adjacent lumbar vertebrae were identified. The abdominal probe was placed in a sagittal subphrenic position just above the iliac crest. The probe was slid anteromedially to poster laterally to identify the largest visible transverse diameter. Three measurements were taken, and their average was used. Measurements were considered “reliable” when: (a) all anatomical boundaries were clearly identified; and (b) no measurement deviated by >5 mm from the others. The psoas muscle thickness-to-height-ratio (PMTH) (mm/m) was then calculated (Figure 1).

**Figure 2:**
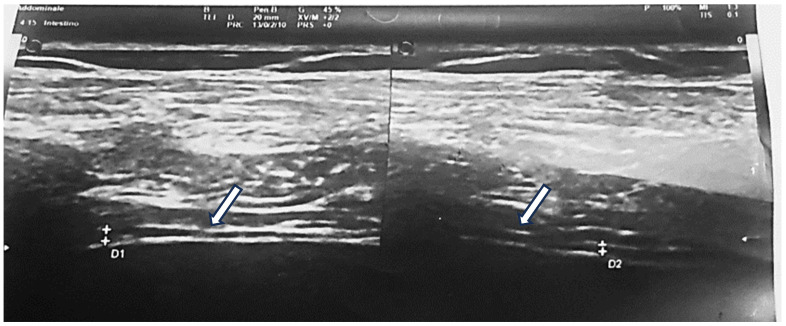
Diaphragm was evaluated at the mid-axillary line on the right side, at the level of either the 8th or the 9th intercostal space depending upon the clearest image, in a 30° head up supine position, the probe held perpendicular to the diaphragm, for muscle thickness.

Diaphragm (DM): The diaphragm was assessed at the right mid-axillary line, at the 8th or 9th intercostal space, in a 30° head-up supine position. Measurements were obtained perpendicular to the diaphragmatic plane to assess muscle thickness at both end-expiration and maximal inspiration. Additional measurements were taken in the apposition zone (between lung and liver on the right, and lung and spleen on the left) in the 9th–11th intercostal spaces, 0.5–2 cm above the costophrenic angle. Diaphragmatic expansion was calculated, and a diaphragm-to-height ratio (mm/m) was derived (Figure 2).

### 2.2. Statistical Analysis

All statistical analyses were performed using *StataMP 17* software (USA). Continuous variables were expressed as medians and interquartile ranges (IQR), unless otherwise specified. Comparisons between groups were performed using nonparametric tests.

Cohen’s kappa statistic was applied to evaluate the degree of agreement between ultrasound indices and sarcopenia diagnosis. Correlations between the psoas-to-height ratio (IP) and diaphragm-to-height ratio (DM) were assessed using Pearson’s correlation coefficient and Bland–Altman analysis.

Categorical variables were compared using the Chi-squared test. Continuous variables were analysed with Student’as *t*-test for independent samples or the Mann–Whitney rank-sum test, depending on the distribution of the data.

A logistic regression model was constructed with sarcopenia as the primary outcome. Independent variables included IBD type, nutritional deficiencies, and nutritional supplementation. Potential confounders, such as sex, age, daily metabolic equivalents (METS), and smoking status, were also considered in multivariate analysis. To further assess the robustness of data, a univariate linear regression model was also performed. A two-sided *p*-value < 0.05 was regarded as statistically significant for all analyses.

## 3. Results

A total of 353 IBD patients (167 CD and 186 UC), 33.3% male, with mean age of 53 years were enrolled. Sixty-seven patients (19.0%) reported a current smoking habit. A mean BMI of 24.35 kg/m^2^ was calculated.

Regarding malnutrition, 211 patients (59.7%) were considered at low risk (MUST = 0), 88 subjects (24.9%) at medium risk (MUST = 1), and 54 (15.3%) at high risk (MUST ≥ 2). A SARC-F score ≥ 4 was observed in 57 patients (16.1%) (Table 1).

Among these 57 SARC-F–positive patients, the male-to-female ratio was approximately 1:2. The mean age was 53 years (IQR 28–62), and the median BMI was 24 kg/m^2^ (IQR 22–28). Thirty patients (52.6%) had Crohn’s disease (CD), while 27 (47.4%) had ulcerative colitis (UC).

In CD, SARC-F positive, patients, disease activity assessed by the Harvey–Bradshaw Index (HBI) was classified as mild in 23.3% (7/30), moderate in 60.0% (18/30), and severe in 16.7% (5/30). In UC patients, disease activity according to the Partial Mayo Score was mild in 29.6% (8/27), moderate in 55.6% (15/27), and severe in 14.8% (4/27).

### 3.1. Study Cohort

Demographic and clinical characteristics of the study population are summarized in Table 1.

### 3.2. Accuracy of Psoas Muscle Ultrasound vs. SARC-F Score

Patients with sarcopenia underwent ultrasound examination of the iliopsoas (IP) muscle, with three consecutive measurements performed in each subject. Mean diameters are reported in Table 2.

The overall mean diameter of the IP muscle was 28.40 mm, with values of 28.82 mm in men and 27.02 mm in women. The calculated psoas muscle thickness-to-height ratio (PMTH) was 16.62 mm/m overall, 17.34 mm/m in men, and 15.86 mm/m in women.

**Figure 3:**
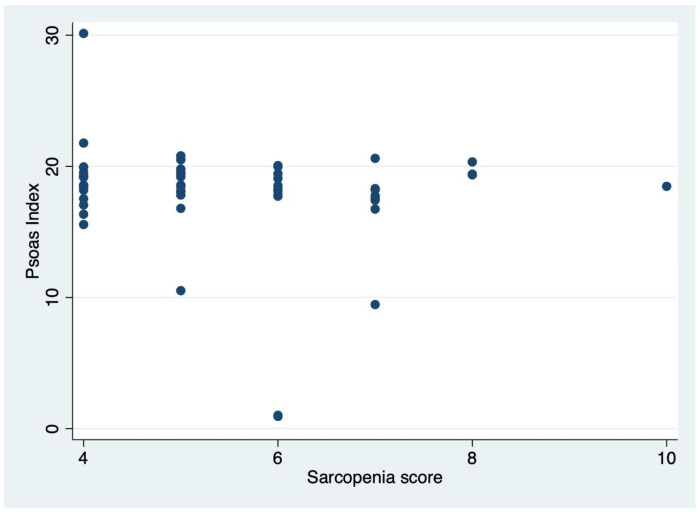
Scatter plot of the correlation between SARC-F score and PMTH index.

These findings are consistent with previously reported thresholds in the literature. Importantly, the PMTH index showed an inverse correlation with the SARC-F score, demonstrating a high degree of diagnostic accuracy (Figure 3).

### 3.3. Accuracy of Psoas Muscle Ultrasound vs. Diaphragm Ultrasound

The diaphragmatic muscle (DM) was assessed at both maximal inspiration and end-expiration. The mean maximal inspiratory diameter was 20.4 ± 5.0 mm, while the mean minimal expiratory diameter was 10.7 ± 3.5 mm. The mean difference between the two diameters was 9.7 ± 3.4 mm, corresponding to diaphragmatic expansion (Table 3).

When corrected for height, the diaphragm-to-height ratio was 0.59 ± 0.21 mm/m. These findings demonstrate good reproducibility and allow comparison with the psoas muscle thickness-to-height ratio (PMTH).

### 3.4. Correlation Between Psoas and Diaphragm Ultrasound Indices

**Figure 4:**
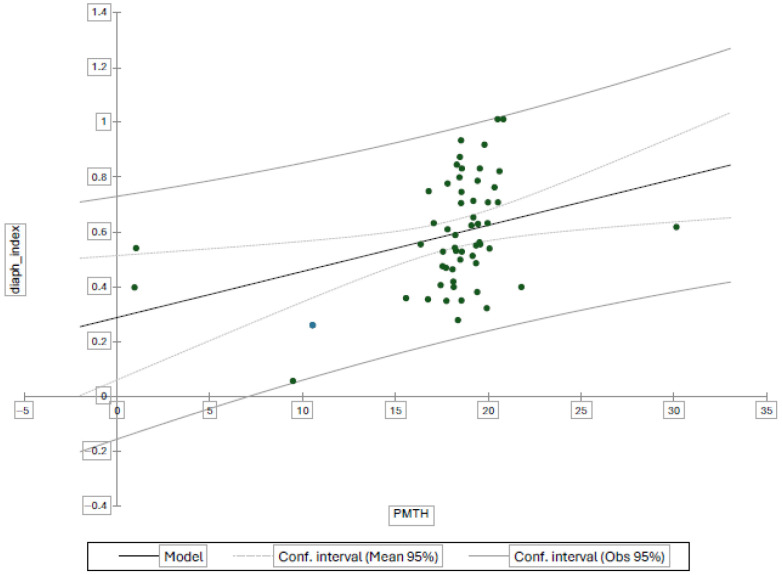
Pearson correlation test between two indexes.

The two ultrasound-derived indices—the psoas muscle thickness-to-height ratio (PMTH) and the diaphragm-to-height ratio—were compared using Pearson’s correlation test (Figure 4). A significant positive correlation was observed, with a Pearson coefficient *r* = 0.357 (*p* < 0.05), indicating that the two ultrasound-derived indices vary in the same direction.

**Figure 5:**
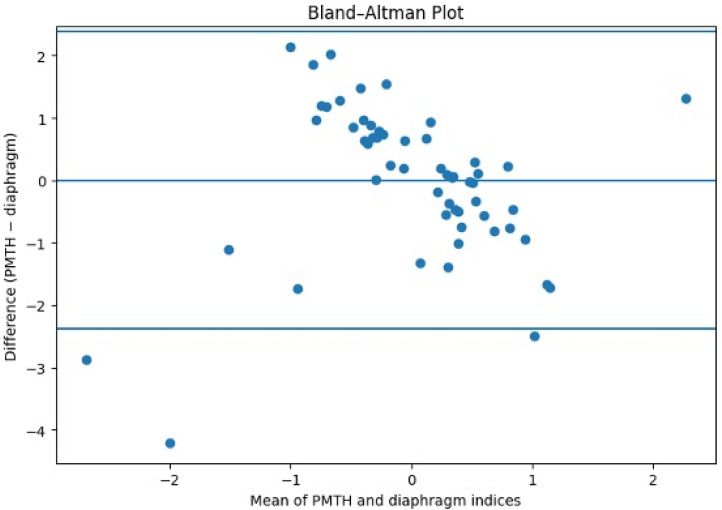
Bland–Altman correlation test between two indexes.

Agreement between the two measures was subsequently evaluated using Bland–Altman analysis (Figure 5). After normalization to allow comparison between indices expressed on different scales, the analysis showed minimal mean bias and a symmetrical distribution of differences across the range of measurements, with no relevant clustering or systematic deviation.

These findings indicate that, although absolute values differ due to the assessment of distinct muscle groups, PMTH and the diaphragm-to-height ratio provide comparable information on muscle status and may be used as alternative ultrasound-derived indices for muscle assessment rather than as directly interchangeable absolute measures.

To further explore this association, a multivariate linear regression model was performed, with the diaphragm-to-height ratio as the dependent variable and the PMTH index, age, and sex as independent variables. The model confirmed a significant linear relationship between the two indices, with the diaphragm index increasing proportionally with the PMTH index (β = 0.018; 95% CI 0.005–0.030; *p* = 0.008). Age and sex were not significantly associated with the diaphragm index (*p* > 0.05).

Additionally, a univariate regression model including only the two echographic indices confirmed the robustness of this correlation (β = 0.017; 95% CI 0.004–0.029; *p* = 0.011).

### 3.5. Correlation of Ultrasound with Disease Activity

Correlations between disease activity scores (partial Mayo for UC and Harvey–Bradshaw Index [HBI] for CD) and US parameters were explored. US-derived indices showed weak-to-moderate correlations with disease activity.

Moreover, US indices were associated with impaired quality of life, as measured by the IBDQ questionnaire (Pearson χ^2^ = 36.41).

Nutritional screening with the MUST identified heterogeneous risk profiles within the study cohort: 16 patients (28.1%) were at low risk, 21 (36.8%) at medium risk, and 19 (33.3%) at high risk of malnutrition. Notably, all high-risk patients were sarcopenic. Among patients with sarcopenia, 18 (66%) were at nutritional risk, but only 6 (22%) were classified as high risk.

Based on ultrasound-derived cut-offs, the prevalence of sarcopenia was estimated at 60% using the RA measurement and 69% using the USMI measurement, confirming the clinical value of ultrasound screening.

## 4. Discussion

This prospective study evaluated the diagnostic accuracy of US for assessing sarcopenia in patients with IBD. The diagnosis of sarcopenia is crucial for gastroenterologists and nutritionists in IBD management as this condition could be a predictor of morbidity and mortality regardless of the underlying disease. A prompt identification addresses nutritional interventions, including adequate protein intake and tailored nutritional support, often combined with physical exercise, in mitigating muscle loss and reducing the metabolic and functional consequences associated with sarcopenia. Importantly, sarcopenia represents a potentially modifiable condition.

Our findings indicate that muscle US is a reliable, rapid, and accurate method for identifying sarcopenia in this population.

In this cohort, we examined US-derived measurements of the psoas and diaphragm muscles. Both indices showed significant correlations with the SARC-F questionnaire, a widely used clinical screening tool for sarcopenia. Inter- and intra-rater reliability was excellent (ICC > 0.95), demonstrating strong reproducibility across operators and repeated assessments. The psoas muscle index correlated strongly with SARC-F–defined sarcopenia, whereas the diaphragm index demonstrated a moderate but consistent association. Given the technical difficulty of obtaining precise psoas measurements, our results highlight the potential value of diaphragm ultrasound as a simpler, more accessible proxy for sarcopenia detection. Early identification of sarcopenia in IBD patients carries important clinical implications. Prompt detection may increase awareness of muscle loss, allow timely nutritional and rehabilitative interventions, and ultimately improve patient outcomes. Beyond its diagnostic and instrumental value, the identification of sarcopenia has important clinical implications.

Notably, US demonstrated greater sensitivity than SARC-F, supporting its integration as a frontline screening tool in routine clinical practice. Patients with IBD are particularly vulnerable to sarcopenia and malnutrition due to chronic inflammation, impaired nutrient absorption, reduced intake, and the catabolic impact of corticosteroid therapy. Both conditions are known to adversely affect disease course and quality of life. Our findings therefore reinforce the importance of incorporating routine muscle assessment into IBD care pathways. Therefore, tools that facilitate the early detection of muscle impairment may contribute to timely interventions and improved patient outcomes This study has several strengths, including its prospective design and its direct comparison of ultrasound indices with established clinical and functional measures. Nevertheless, certain limitations warrant consideration. The sample size was relatively small, but consistent with the epidemiological data on the prevalence of sarcopenia in the IBD population, the study was conducted at a single center, and follow-up duration was limited. Additionally, we did not incorporate advanced ultrasound-derived quality parameters recommended by the SARCUS working group (e.g., pennation angle, fascicle length, echo-intensity, stiffness, contraction potential, microcirculation) [18], which may enhance diagnostic precision in future investigations.

## 5. Conclusions

In conclusion, this exploratory study suggests that ultrasound-based assessment of muscle mass may represent a feasible and informative approach for evaluating muscle status in patients with inflammatory bowel disease. The observed associations between ultrasound-derived muscle indices, disease activity, and nutritional risk indicate a potential link between muscle impairment and the clinical severity of IBD.

While these findings should be interpreted with caution, they support the hypothesis that ultrasonography could contribute to a more comprehensive evaluation of patients at risk of sarcopenia. Further prospective studies involving larger, multicenter cohorts and standardized imaging protocols are warranted to confirm these preliminary observations and to define clinically meaningful thresholds for ultrasound-based muscle assessment.

## Figures and Tables

**Table 1:** Baseline characteristics of the “study cohort”.

Variable	Total (*n* = 353)
Sex, *n* (%)	
Male/Female	33.3/66.7
Age, years	
Median (range)	53 (28–62)
BMI, kg/m^2^	
Median (IQR)	24.3 (22–28)
IBD type, *n* (%)	
Crohn’s disease (CD)	186 (52.6)
Ulcerative colitis (UC)	167 (47.4)
Disease activity	
Partial Mayo score (UC), moderate–severe (2–3), %	29.1/43.1
HBI mild/moderate-severe (CD), %	12.6/87.4
Malnutrition risk (MUST), *n* (%)	
MUST 0/1/≥2	59.7/25.0/15.3
SARC-F score ≥ 4, *n* (%)	57 (16.1)
Reduced quality of life (IBDQ < 209), *n* (%)	124 (35.1)
Biological therapy, *n* (%)	279 (79.0)
Corticosteroid therapy, *n* (%)	42 (12.0)
Current smoking habit, *n* (%)	67 (19.0)

**Table 2:** Ileo-psoas diameter on three consecutive measurements.

Measurement	Mean Diameter (mm) ± SD	Male (mm) ± SD	Female (mm) ± SD
First	28.38 ± 3.00	29.87 ± 3.17	28.28 ± 2.95
Second	28.48 ± 3.05	29.52 ± 3.12	28.46 ± 3.05
Third	27.39 ± 3.25	28.50 ± 3.19	27.33 ± 3.32
Overall mean	28.40 ± 3.07	28.82 ± 3.15	27.02 ± 3.07
Mean diameter/height (mm/m)	16.62 ± 4.14	17.34 ± 3.41	15.86 ± 4.50

**Table 3:** The diaphragm’s diameter measures, both in inspiration and expiration.

Parameter	Overall (Mean ± SD)	Male (Mean ± SD)	Female (Mean ± SD)
Maximum diameter (mm)	20.4 ± 5.0	20.3 ± 5.4	20.5 ± 4.9
Minimum diameter (mm)	10.7 ± 3.5	10.6 ± 3.6	10.7 ± 3.4
Difference max–min (mm)	9.7 ± 3.4	9.7 ± 3.2	9.8 ± 3.6
Difference/height (mm/m)	0.59 ± 0.21	0.57 ± 0.20	0.61 ± 0.22

## Data Availability

The data presented in this study are available on request from the corresponding author. The data are not publicly available due to ethical restrictions.
